# Long Non-Coding RNA: Dual Effects on Breast Cancer Metastasis and Clinical Applications

**DOI:** 10.3390/cancers11111802

**Published:** 2019-11-16

**Authors:** Qi-Yuan Huang, Guo-Feng Liu, Xian-Ling Qian, Li-Bo Tang, Qing-Yun Huang, Li-Xia Xiong

**Affiliations:** 1 Department of Pathophysiology, Basic Medical College, Nanchang University, Nanchang 330006, China; 6301616056@email.ncu.edu.cn (Q.-Y.H.); 6302615051@email.ncu.edu.cn (X.-L.Q.); lb525912836@163.com (L.-B.T.); 401442718029@email.ncu.edu.cn (Q.-Y.H.); 2 Second Clinical Medical College, Nanchang University, Nanchang 330006, China; 3 First Clinical Medical College, Nanchang University, Nanchang 330006, China; 6302615053@email.ncu.edu.cn; 4 Jiangxi Province Key Laboratory of Tumor Pathogenesis and Molecular Pathology, Nanchang 330006, China

**Keywords:** lncRNA, breast cancer, metastasis, cancer stem cell, angiogenesis, chemotherapy resistance, prognosis

## Abstract

As a highly heterogeneous malignancy, breast cancer (BC) has become the most significant threat to female health. Distant metastasis and therapy resistance of BC are responsible for most of the cases of mortality and recurrence. Distant metastasis relies on an array of processes, such as cell proliferation, epithelial-to-mesenchymal transition (EMT), mesenchymal-to-epithelial transition (MET), and angiogenesis. Long non-coding RNA (lncRNA) refers to a class of non-coding RNA with a length of over 200 nucleotides. Currently, a rising number of studies have managed to investigate the association between BC and lncRNA. In this study, we summarized how lncRNA has dual effects in BC metastasis by regulating invasion, migration, and distant metastasis of BC cells. We also emphasize that lncRNA has crucial regulatory effects in the stemness and angiogenesis of BC. Clinically, some lncRNAs can regulate chemotherapy sensitivity in BC patients and may function as novel biomarkers to diagnose or predict prognosis for BC patients. The exact impact on clinical relevance deserves further study. This review can be an approach to understanding the dual effects of lncRNAs in BC, thereby linking lncRNAs to quasi-personalized treatment in the future.

## 1. Introduction

Nowadays, breast cancer (BC) is one of the most common cancers on a global scale, and it is the main cause of cancer death in women. In 2018, the number of newly diagnosed female BC cases worldwide reached 2.1 million, accounting for nearly a quarter of female cancer cases [1]. Metastasis is responsible for most cases of cancer mortality and relies on an array of processes, including the bilateral transition between epithelial-to-mesenchymal transition (EMT) and mesenchymal-to-epithelial transition (MET), promotion of cancer cell invasion, migration, stemness and metastasis, inhibition of cancer cell proliferation, anoikis, and post metastatic angiogenesis. Therefore, molecular mechanisms that affect any of these processes may be involved in regulating tumor metastasis.

Ever since the definition of four subtypes of BC (luminal A, luminal B, human epidermal growth factor receptor 2 positive (HER-2+), and basal-like), the clinical treatment for BC patients has become more specific and individualized. An increasing amount of mechanism research has made it possible to individualize treatment and achieve a better prognosis for patients. For example, in HER-2 overexpression patients, the successful application of anti-HER-2 drugs commendably demonstrates the advantages of the latest achievements in molecular science of basic medicine [2]. Triple negative breast cancer (TNBC) is defined by the negative expression of estrogen receptors (ER), progesterone receptors (PR), and the lack of expression of HER-2 [3]. Clinically, TNBC has high invasiveness and the metastasis rate is also abnormally high [4]. A lack of receptor expression leads to a low chemotherapy response rate and poor efficiency of targeted therapy. Until now, there has been no clear and effective molecular targeted therapy for TNBC [5]. Previous studies have demonstrated the crosstalk between tumor metastasis and therapy resistance in various cancers, including BC; among them, EMT regulation has a crucial role [6]. Additionally, EMT programming in cancer cells enables the remodeling of the extracellular matrix to break the dormancy of relapse-initiating cancer stem cells (CSCs) [7].

According to the encyclopedia of DNA elements (ENCODE) project, more than 80% of the human genome is transcribed into biochemically functional non-coding RNAs (ncRNAs) [8]. Transfer RNAs (tRNAs), ribosomal RNAs (rRNAs), microRNAs (miRNAs), small nuclear RNAs (snRNAs), and long non-coding RNAs (lncRNAs) are all types of ncRNAs [9]. LncRNAs are a class of ncRNA with a length over 200 nucleotides and can be divided into five categories according to the position of their DNA fragments in the genome, including bidirectional, antisense, intergenic, intronic, and sense lncRNAs [10].

As part of the ENCODE consortium, GENCODE (version 32) annotated 17,910 lncRNA genes and 48,351 lncRNA transcripts [11]. Among them, previous studies have indicated as many as 60,000 lncRNAs in humans and other mammals [12]. The discovery of numerous lncRNA transcripts has dramatically altered our understanding of cell biology, especially the biology of underexamined diseases such as cancer. Currently, many studies have investigated the association between lncRNAs and cardiovascular disease [13,14], neurological diseases [15,16], diabetes [17], cancer [18], and more. LncRNAs function through a variety of molecular mechanisms, such as acting as scaffolds for ribonucleoprotein (RNP) complexes, decoys for transcriptional factors or miRNAs, RNA interference, targeting of transcriptional factors or chromatin modifier proteins to specific genomic loci, and transcriptional regulation in cis or trans [19]. Interestingly, a number of studies have proposed that lncRNAs could inactivate miRNAs through a “sponge” effect, namely by sequestering miRNAs from their target mRNAs as a competing endogenous RNA (ceRNA) [20,21]. This interaction also exists during cancer progression [22,23]. In addition to basic research, the clinical application of lncRNAs is also an emerging research field [24]. LncRNAs are ideal diagnostic biomarkers and therapeutic targets. As of yet, due to the lack of basic clinical research, lncRNAs have not been applied in commercial diagnostic tests.

In this study, we reviewed the latest progress in this area and found that lncRNAs not only have a role in regulating the aggressiveness of BC cells but also have an effect on the distant metastasis and therapy resistance in BC patients. To our surprise, we found that lncRNAs also have a role in regulating the stemness of cancer stem cells and tumor angiogenesis, affecting cancer metastasis. In addition, by reviewing a number of studies, we discuss whether and how lncRNAs facilitate the diagnosis and prognosis prediction and improve the specificity for therapy of BC by acting as biomarkers. A considerable number of studies have uncovered the effect of lncRNAs on BC metastasis via various signaling pathways, which is also a focus of this review.

## 2. LncRNAs That Enhance Aggressiveness of BC Cells

### 2.1. LncRNAs That Promote Invasion and Migration of BC Cells

#### 2.1.1. DANCR

Previous studies have identified lncRNA differentiation antagonizing non-protein-coding RNA (lncRNA DANCR) associated with hepatocellular carcinoma, colorectal cancer, and BC, especially in TNBC [25,26,27]. Knockdown of DANCR inhibited BC cell invasion and migration. Mechanistically, DANCR downregulation impaired the expression of CD44 and ATP binding cassette subfamily G member 2 (ABCG2) through enhancement of the zeste homolog 2 (EZH2) [27]. EZH2 is a part of the polycomb repressive complex 2 (PRC2) that facilitates target gene silencing through trimethylation of histone 3 lysine 27 (H3K27) residues [28]. DANCR suppression could have a negative effect on EZH2 and PRC2-mediated H3K27-trimethylation at the epigenetic level [29]. Another potential mechanism was identified by bioinformatics analysis, which confirmed the DANCR/miRNA-216a-5p axis in TNBC cells, and DANCR knockdown resulted in inhibited cell invasion and decreased expression of Nanog, SRY-box containing gene 2 (SOX2), and octamer-binding transcription factor-4 (OCT4) [30].

#### 2.1.2. H19

LncRNA H19 has been identified to be functionally associated with many biological processes, such as cell proliferation, invasion, and apoptosis of tumors, including in BC [31,32]. DNA hypermethylation is involved in BC carcinogenesis and cell survival, and is initiated by abnormal expression of DNA methyltransferases (DNMTs), such as DNMT1, DNMT3a, and DNMT3b [33,34,35]. Evidence has indicated that H19 is aberrantly upregulated in BC and promotes invasion of BC cells via the miR-152/DNMT1 axis, and such outcomes can be reversed by miR-152 overexpression and DNMT1 knockdown [36]. H19 and insulin-like growth factor 2 (IGF2) usually jointly accelerate cancer development of mammary and pulmonary tumors [37,38]. At the H19/IGF2 locus, Vennin et al. discovered a newly conserved lncRNA antisense to H19 gene—H91 [39]. H91 promotes IGF2 gene expression through a novel promoter named Pm [40]. In-vitro and in-vivo studies have established that over-expressed H91 disturbs epigenetic modifications of H19 and IGF2 to promote BC development as an oncogene [39].

#### 2.1.3. HOTAIR

Homeobox transcript antisense RNA (HOTAIR) is closely related to BC size, advancement, and extent of metastasis [41,42]. Years ago, it was speculated that HOTAIR might promote tumor aggressiveness through the upregulation of vascular endothelial growth factor (VEGF) and matrix metalloproteinase (MMP) and EMT-related genes [43]. Knockdown of HOTAIR inhibits the migration and invasion ability of BC cells via the P53/protein kinase B (AKT)/c-Jun N-terminal kinase (JNK)/MMPs signaling pathway [44] and the AKT/mammalian target of the rapamycin (mTOR) signaling pathway [45]. Currently, based on the bidirectional interaction between lncRNAs and miRNAs, researchers are trying to investigate more signaling networks involving miRNAs. HOTAIR could act as a sponge for miR-204 and significantly mitigates cell migration and vasculogenic mimicry through the HOTAIR/miR-204/FAK axis in TNBC [46].

#### 2.1.4. LINC00152

LncRNA LINC00152, an 828-bp lncRNA that is located on chromosome 2p11.2, was initially defined as differentially hypomethylated during hepatocarcinogenesis [47]. LINC00152 is highly expressed in various types of BC tissues, promoting invasion and migration of cancer cells [48,49,50]. With the knockdown of LINC00152, many studies observed inhibited migration and invasion of MDA-MB-231 cells and MCF-7 cells [49,50]. In agreement with findings in TNBC cell lines, LINC00152 is highly expressed in TNBC tissues and mechanistically LINC000152 induces tumorigenesis by inactivation of the tumor-suppressive breast cancer gene 1 (BRCA1)/gene of phosphate and tension homology deleted on chromosome ten (PTEN) signaling [48]. In ER-positive cancer cells, Hu et al. cast light not only on the positive effect of LINC00152 on one of the cellular preconditions for cancer metastasis, EMT, but also on chemo-resistance to doxorubicin (DOX) [49].

#### 2.1.5. LINC00461

LncRNA LINC00461 is a novel tumor promoter in BC and is transcribed from a gene located at an intergenic region of human chromosome 5 [51]. Over-expression of LINC00461 results in upregulated expression of vimentin, E-cadherin, and ZEB1 [52,53,54]. Additionally, up-regulation of LINC00461 accelerates BC cell migration and invasion through the miR-30a-5p/integrin β3 axis [53]. These findings provide a basis for the association of LINC000461, EMT, and aggressive phenotypes of human cancer.

#### 2.1.6. NEAT1

Transcriptome sequencing and next-generation sequencing files have shown that nuclear-enriched abundant transcript 1 (NEAT1) is one of the highest expressed lncRNAs in BC [55,56]. Across the four subtype-related ceRNA networks, NEAT1 has specific roles in each subtype through competing with diverse mRNAs [56]. These results hint at a strong connection between oncogenic effects of NEAT1 and BC. The latest studies suggest NEAT1 has a crucial role in promoting the growth, migration and invasion capacity of BC cells [57]. Despite the lack of in-vivo research with clinical BC samples, NEAT1 has been verified as an miR-448 sponge to enhance zinc finger E-Box binding homeobox 1 (ZEB1) expression, and this phenomenon can be reversed by an miR-448 mimic in vitro [58]. In triple-negative breast cancer, NEAT1 confers oncogenic effects through modulating chemoresistance and cancer stemness [59].

#### 2.1.7. LINC01857

As a sharply up-regulated lncRNA in BC tissue, previous studies have noted that LINC01857 is usually associated with poor prognosis of BC patients [60]. The cAMP response element-binding protein binding protein (CREBBP) is an acetyltransferase that is commonly considered a tumor suppressor in BC [61,62]. Knockdown of LINC01857 inhibits H3K27Ac and cAMP response element-binding protein 1 (CREB1) transcription via reducing the enrichment of CREBBP in the CREB1 promoter region. Overexpression of CREB1 in MCF-7 cells reverses the inhibition effect of LINC01857 knockdown, leading to larger quantities of migration and invasion cells [60].

### 2.2. LncRNAs That Promote Distant Metastasis of BC Cells

#### 2.2.1. H19

In 2017, Zhou et al. isolated BC cells from the primary mammary tumor, circulation, and metastatic lesions in the lungs of TA2 mice and found that H19 is an essential key factor in several tumor metastasis procedures. In cells isolated from plasma blood, H19 acts as an endogenous sponge by directly binding to let-7b. Subsequently, the binding of let-7b and cytohesin 3 (CYTH3; one of ArfGEFs) activates adenosine 5′-diphosphate (ADP) ribosylation factor (ARF) and EMT [63]. Contrarily, in cells isolated from the primary tumor and lung metastasis tumor, H19 regulates GIT2 via sponging miR-200b/c and promotes G protein-coupled receptor kinase interacting protein 2 (GTI2; one of ArfGAPs) expression through inactivating ARF [63]; thus, MET took place in order to accelerate successful metastatic colonization of a secondary organ [64,65]. Matouk et al. revealed in their research that hypoxia-induced EMT is associated with strong induction of both H19 and miR-675, and the H19 gene is highly expressed in common metastatic sites regardless of the tumor’s primary origin [66]. Therefore, we suggest H19 is a tumor-promoting lncRNA that endows distant metastatic potential to BC cells.

#### 2.2.2. HOTAIR

In recent years, HOTAIR has been one of the most well-studied lncRNAs. Alongside invasion and migration of BC cells, HOTAIR participates in metastasis of BC as well. Mounting evidence has revealed the positive correlation between high circulating HOTAIR and lymph node metastasis, as well as distant metastasis of BC [67].

Epigenetic signaling from the tumor microenvironment regulates the switch from dormancy to metastatic growth [68]. Apart from studies focusing on cross talks in the BC cell itself, the latest emerging studies have investigated the roles of lncRNAs in the tumor microenvironment. By establishing an orthotopic mouse model of MDA-MB-231 cells, previous studies have explored a novel transforming growth factor-beta1 (TGF-β1)/cyclin-dependent kinase 5 (CDK5)/HOTAIR/H3K27 signaling that could promote tumor growth, lymph node metastasis, and breast-to-lung metastasis [69]. Additionally, emerging evidence from laminin-rich extracellular matrix-based three-dimensional organotypic culture (lrECM 3D) has suggested the presence of epigenetic regulation of gene expression by extracellular matrix (ECM) signaling in cancer cells [70,71]. Interestingly, HOTAIR expression exhibits robust induction in lrECM 3D over that in 2D culture, and such an induction partly relies on bromodomain containing 4 (BRD4) and the canonical ECM signaling pathway, namely integrins and Src kinase [72]. This validates the significant observations of Gupta et al., who suggested conventional 2D cultures which lacked critical factors that stimulated the expression of HOTAIR and promoted cancer metastasis very early [73].

#### 2.2.3. HIF1A-AS2

Hypoxia-inducible factor 1 alpha-antisense RNA 2 (HIF1A-AS2) may be a latent tumor promotor in TNBC patients. In vitro, HIF1A-AS2 obviously depresses TNBC cell migration and invasion; in 86 TNBC cases, high expression of HIF1A-AS2 was associated with more lymph node metastasis, distant metastasis, and unfavorable histological grade [74]. Previous studies have found even higher plasma levels of HIF1A-AS2 expression in BC cases with lymph node metastasis [75].

#### 2.2.4. MALAT1

The nuclear lncRNA metastasis-associated lung adenocarcinoma transcript 1 (MALAT1) is among the most conserved and highly abundant lncRNAs in normal tissues, suggesting that it may have vital biological implications [76]. When it comes to BC metastasis, overwhelming previous findings indicate its increased expression is associated with relapse and metastatic progression in BC. Up-regulation of MALAT1 in the tissue and serum of BC, generated by different strategies, has been reported by independent groups [77,78,79]. This is one novel epigenetic mechanism by which MALAT1 facilitates a pro-metastatic phenotype in BC by trans-regulating EEF1A1 [80]. Li et al. overexpressed MALAT1 and found this promoted lipopolysaccharide (LPS)-induced invasion and metastasis of human and mouse mammary tumor cells [81]. Moreover, a novel regulation mechanism of MALAT1 in BC cells was presented by more recent research. They suggested that hypoxia might induce the specific chromatin interactions and increases MALAT1 expression as well as its antisense strand TALAM1 in BC cells [82]. An in-vivo study showed that Malat1^−/−^ mice exhibited a significant reduction in lung macrometastases and a significantly decreased metastatic burden. Moreover, knockdown of Malat1 results in mammary tumor differentiation [83]. Similar conclusions can be drawn from in-vitro and in-vivo studies on TNBC. Knockdown of MALAT1 impaired TNBC growth and metastasis via the miR-1/slug axis [79] and MALAT1/miR-129-5p axis [84].

## 3. LncRNAs That Attenuate Aggressiveness of BC Cells 

### 3.1. LncRNAs That Inhibit Invasion and Migration of BC Cells

#### 3.1.1. GAS5

Growth arrest-specific 5 (GAS5) is a noncoding gene that was initially isolated from mouse NIH 3T3 cells [85]. The expression level of GAS5 is significantly lower in BC in younger cases [86]. Additionally, studies have demonstrated the negative correlation between GAS5 expression and malignancy of different cell lines of BC [87]. GAS5 could function as an endogenous sponge that sequesters miR-21, resulting in reciprocal repression of miR-21 and GAS5. This interaction relies on the putative miR-21-binding site at exon 4 of GAS5 and the RNA-induced silencing complex (RISC) [87]. Additionally, GAS5 inhibits TNBC cell invasion through the miR-196a-5p/forkhead box protein O1 (FOXO1)/phosphatidylinositol 3-kinase (PI3K)/AKT axis, which starts with competitive binding of GAS5 to miR-196a-5p [88].

#### 3.1.2. MT1JP

The lncRNA metallothionein 1J pseudogene (MT1JP) is located on chromosome 16 in a cluster that consists of several homologous protein-coding genes in the metallothionein family. It was first reported as a tumor suppressor that modulates the p53 protein expression level, thereby regulating the p53-related signaling pathway [89].

In terms of BC, one recent study presented the first evidence that MT1JP overexpression markedly inhibits invasion and enhances cisplatin sensitivity of BC cells, which is coincident with gastric cancer results. Mechanistically, MT1JP competitively binds to miR-24-3p and inhibits the Wnt/β-catenin signaling pathway [90]. More importantly, down-regulated MT1JP expression in BC patients indicates a better prognosis.

#### 3.1.3. NEF

A considerable number of studies have observed the down-regulated expression of lncRNA-neighboring enhancer of FOXA2 (NEF) as a tumor suppressor in various types of cancers, for instance, cholangiocarcinoma [91], non-small-cell lung cancer [92], and hepatocellular carcinoma [93]. BC is no exception. Previous in-vitro cell experiment results have indicated that NEF might inhibit TNBC cell migration and invasion by downregulating miRNA-155, and overexpression of miRNA-155 significantly attenuates the inhibitory effects of NEF overexpression on cancer cell migration [94].

#### 3.1.4. NKILA

In various cancer types, evidence indicates that TGF-β-induced EMT is nuclear factor kappa-B (NF-κB)-dependent. LncRNA NKILA is a newly discovered lncRNA that functions as a tumor suppressor in BC metastasis [95,96]. In BC, recent research has linked the negative feedback regulation of lncRNA NKILA with NF-κB activation and TGF-β-induced EMT, thus indicating NKILA may inhibit the breast EMT process of BC cells, thus retarding metastasis. Mechanistically, NKILA can suppress several NF-κB target genes by directly binding to the NF-κB/IκB complex [97]. Furthermore, there is a positive correlation between NKILA and E-cadherin expression level, and the long-term survival of BC patients [96].

#### 3.1.5. LET

LncRNA LET is a newly discovered molecule involved in the invasion and migration of BC. Despite the overall expression of LET in BC tissue and cell lines being down-regulated, LET expression differs in various BC cell lines. LET expression is lower in MDA-MB-231 (BC cell line, triple negative) than MCF-10 (non-tumorigenic epithelial breast cell line), which suggests the negative association between the LET expression level and the acquisition of tumorigenesis abilities of BC cells. Additionally, over-expressing LET increases E-cadherin and decreases N-cadherin and vimentin expression, and thereby represses EMT of BC cells [98].

#### 3.1.6. TFAP2A-AS1

Transcription factor AP-2 alpha-antisense RNA 1 (TFAP2A-AS1) may be a novel tumor suppressor in BC. Transwell assay showed a remarkable decrease of invasive cell numbers in MCF-7 and MDA-MB-231 cells transfected with TFAP2A-AS1. Mechanistically, TFAP2A-AS1 sequesters the miR-933-inhibiting Smad signaling pathway by downregulating drosophila mothers against decapentaplegic family member 2 (SMAD2). An in-vivo study showed that TFAP2A-AS1-overexpressed MCF-7 cells injected in nude mice ended up with a significantly smaller tumor volume and weight [99].

#### 3.1.7. LncKLHDC7B 

With knockdown of LncKLHDC7B, Beltran-Anaya et al. confirmed an inverse association between the unique LncKLHDC7B overexpression and migration and invasion in the immunomodulatory subtype of TNBC [100]. However, they did not investigate the potential mechanism further. To understand the clinical implications of LncKLHDC7B expression, they analyzed public data in 2012 and 2016 [101,102,103] and found that lower LncKLHDC7B expression was related to lower survival and an increased risk of a recurrent or metastatic event.

### 3.2. LncRNAs That Inhibit Distant Metastasis of BC Cells

#### 3.2.1. MALAT1

On the one hand, as we discussed before, increased expression of MALAT1 is associated with relapse and metastatic progression in breast cancer. On the other hand, a few studies surprisingly provide new insight into MALAT1 as a metastasis-suppressing lncRNA in BC. Several studies have found down-regulated expression of MALAT1 in BC [104,105]. Kwok et al. suggested that the PTEN-microRNA-MALAT1 axis may promote tumorigenesis and demonstrated the first evidence that MALAT1 possesses novel tumor-suppressive capabilities in BC [106]. A similar case was found by establishing both genetically engineered mouse models and xenograft models; Kim et al. observed that metastasis was specifically induced by somatic knockout of Malat1. Mechanistically, MALAT1 prevents prometastatic transcription factor TEA domain family member 2 (TEAD) from associating with its co-activator Yes-associated protein (YAP) [104].

Collectively, we cannot conclude whether MALAT1 is a tumor-promoting or suppressing lncRNA; numerous looping events remain to be discovered to explore the possible explanation for the dual effects of MALAT1 in BC.

#### 3.2.2. MEG3

LncRNA maternally expressed gene 3 (MEG3) has been implicated in tumorigenesis and progression of BC, and the mechanisms in most cases are associated with DNMT of MEG3. It has been demonstrated that miR-506 could target DNMT1 and DNMT3b to retard tumor development [107]. Downregulating miR-506 increases the methylation level of MEG3 promoter and inhibits MEG3 expression via the miR-506/SP3/SP1/DNMT1/MEG3 axis in human BC cell lines, resulting in attenuation of metastasis of MCF-7 and MDA-MB-231 cells [108]. Accordingly, it is plausible to speculate that both miR-506 and MEG3 function as tumor suppressors in BC, which is coincident with previous results [109,110]. Additionally, treatment of DNA methylation inhibitor (5′-Aza-2′-deoxycytidine) may partly reverse the signaling and retard tumor development [111].

#### 3.2.3. NLIPMT

Jiang et al. identified a hitherto uncharacterized lncRNA called novel lncRNA, inhibiting proliferation and metastasis (NLIPMT) in BC. An in-vitro study and xenograft study showed that over-expression of NLIPMT inhibited cell motility, growth, and metastasis of BC. Mechanistically, downregulated NLIPMT accelerated proliferation and restored motility of BC cells by promoting the expression of glycogen synthase kinase 3β (GSK3β) and EMT proteins [112]. Interestingly, according to previous studies, GSK3β has dual effects in different types of cancer. On the one hand, evidence indicates that GSK3β functions as a tumor suppressor in the breast [113,114,115]. On the other hand, a tumor-promoting effect of GSK3β has been indicated in colon [116] and pancreatic cancers [117]. Therefore, the conflict of GSK3β’s functions might disturb the effect of NLIPMT in different types of cancer.

#### 3.2.4. XIST

The lncRNA X inactive specific transcript (XIST) is a potential tumor suppressor in cancer [118,119]. A luciferase reporter assay verified that XIST targets the miR-155/CDX1 axis, and overexpression of XIST remarkably inhibits BC cell migration and invasion [120]. Xing et al. demonstrated how the overexpression of XIST in BC cells significantly attenuates its metastatic ability to the brain from two different aspects. Firstly, a pathway screening based on gene set enrichment analysis (GSEA) uncovered that XIST activates the c-Met signaling pathway by activation of moesin (MSN) on the X chromosome. MSN, a member of the ERM (ezrin, radixin, and moesin) protein family, participates in the maintenance of epithelial integrity [121]. Loss of c-Met decreases the transmigration abilities of BC cells and suppresses the breast-to-brain metastatic abilities in vivo [122]. Secondly, considering activated microglia can modulate neurodegenerative diseases and tumor progression under pathological conditions [123], they also investigated the role of XIST in microglia. Interestingly, XIST-downregulated BC cells could secrete exosomal miR-503 to reprogram microglia from the M1 (tumor-suppressive) into the M2 (tumor-promoting) phenotype, causing local immune suppression [122].

## 4. The Regulation of LncRNAs in the Stemness of BC Cells

The cancer stem cell theory indicates that cancers are sustained by tumor-initiating cells, and researchers initially termed these cancer stem cells, with distinct phenotypes and a high tumorigenic potential [124]. CSCs are the “seeds” of tumor metastasis and recurrence, and have a limitless and high invasion and migration capacity [125]. The subpopulation of CSCs will express specific biomarkers, giving rise to metastasis; therapy resistance; and the recurrence of cancers such as CD 26, CD133 and CD44v6 [126,127].

Currently, with further research emerging on lncRNAs, many researchers have suggested that lncRNAs are required for stemness maintenance of cancer cell lines [128]. However, whether and how lncRNAs have a role in breast cancer stem-like cells (BCSCs) remains unclear. Discussing the potential mechanism of lncRNA function may contribute to developing novel therapeutic approaches. Herein, we review our current understanding of the cell-biological mechanisms that regulate BC metastasis, with BCSCs and lncRNAs as the pivotal objects.

EMT is a cellular process that is not only strongly correlated with tumor metastasis, but also with CSC [129]. LncRNAs have been demonstrated to play vital roles during EMT, and some of these molecules also have regulatory roles in the proliferation of CSC [130], although their functions are still unclear in BC. Current research indicates that T-cell leukemia 1 upstream neural differentiation-associated RNA (TUNAR, LINC00617) functions as a vital promotor of EMT and causes generation of stem cell properties via activating the transcription of SOX2, thus promoting BC progression and metastasis [131]. LncRNA ES1 (LINC01108) and a hedgehog pathway-associated lncRNA called lncRNA-Hh can enhance the stem cell properties of BC via regulating SOX2 as well [132,133]. ES1 controls the expression of stemness transcription factors in BC cells by regulating the OCT4/SOX2/miR-302 axis [132]; lncRNA-Hh directly targets GAS1 to increase the SOX2 and OCT4 expression, thus promoting the CSCs-like characteristics of Twist-driven EMT cells and Twist-positive BC cells [133]. LncRNAs could also attenuate stemness in BC cells via inhibiting EMT. The study on brain metastasis of BC found that the lncRNA XIST suppressed EMT and the MSN/c-Met axis via MSN-mediated protein stabilization, which leads to the attenuation of stemness in the tumor cells. The negative correlation between RNA of XIST and c-Met expression was also observed in brain metastasis samples from BC patients [122].

LncRNAs can regulate oncogenic transcription factor in stemness properties via sponging miRNAs as a ceRNA. FEZ family zinc finger 1-antisense 1 (FEZF1-AS1) and LINC00511 can promote the BCSC properties via FEZF1-AS1/miR-30a/Nanog and the LINC00511/miR-185-3p/E2F1/Nanog signaling pathway, respectively [134,135]. Contrarily, the tumor-suppressive lncRNA fibroblast growth factor 13-antisense RNA 1 (FGF13-AS1) could act as an endogenous sponge by directly binding to Myc mRNA via binding insulin-like growth factor 2 mRNA binding proteins (IGF2BP), followed by the obstructive combination of IGF2BPs and Myc mRNA [136]. Myc is an oncogenic transcription factor that plays vital roles in glycolysis and stemness properties [137]. Furthermore, a feedback loop between Myc and FGF13-AS1 also participates in regulating these novel suppressive effects of FGF13-AS1 [137]. Impaired function of Myc may explain how FGF13-AS1-inhibited BC cells achieve migration and invasion.

In addition to the effect of lncRNA dysregulation on EMT proteins and oncogenic transcription factors in stemness properties, the latest studies point out that lncRNAs play a role in BCSC maintenance under hypoxia via the tricarboxylic acid cycle [138]. Mechanistically, H19 sequesters miRNA let-7 as a ceRNA, leading to hypoxia-inducible factor-1α (HIF-1α) release and an increase in pyruvate dehydrogenase kinase 1 (PDK1) expression [139]. PDK1, an essential glycolytic enzyme, is correlated with tumor proliferation, metastasis, and poor prognosis [140,141]. Therefore, it is plausible to conclude that H19 may activate PDK1 in BCSC via the let-7/HIF-1 axis under hypoxia, and this mechanism may be required in the development of primary breast carcinomas.

## 5. The Regulation of LncRNAs in the Angiogenesis in BC

Angiogenesis refers to the process of neovessels sprouting from preexisting vessels, as opposed to the vasculogenesis of embryonic fibroblasts and their consequently activating proliferation, invasion, and metastasis of cancer cells [142]. It is well known that the more pathological angiogenesis occurs, the more chances for cancer cells to migrate into the circulatory system and achieve distant metastasis. However, during the last few years, despite many reviews aimed at organizing the mechanism underneath BC angiogenesis, only a few reviews have associated BC angiogenesis with lncRNAs. Here, we review how lncRNAs participate in the angiogenesis of BC, thus further supporting the understanding of their involvement in promoting or inhibiting BC metastasis.

LncRNA MALAT1 is considered an oncogenic regulator of BC, and we have discussed its role in regulating the aggressiveness of BC cells. Vascular endothelial growth factor (VEGF) is the crucial signaling molecule for angiogenesis, acting by regulating proliferation, survival, and migration of the cancer [143]. In terms of BC angiogenesis, up-regulated MALAT1 in BC tissue also shows oncogenic effects in vitro and in vivo through VEGF regulation. Mechanistically, knockdown of MALAT1 significantly promotes angiogenesis of BC through upregulating the expression level of miR-145 in MCF-7 cells and BC tissue [144].

Some tumor-suppressive lncRNAs also exhibit negative regulatory effects on BC angiogenesis. For example, MEG3 not only inhibits the aggressiveness of BC cells, but also inhibits BC angiogenesis via regulating the VEGF or AKT pathway [145,146].

## 6. The Regulation of LncRNAs in Chemotherapy Resistance in BC

The current clinical treatments for BC patients include surgery and postoperative chemotherapy, targeted therapy, or radiation therapy [147]. It is critical to select the chemotherapy regimens wisely, because different types of BC will respond to different regiments and specific drugs. For example, ribociclib plus fulvestrant (hormone receptor-positive/HER-2+) [148]; trastuzumab, neratinib and lapatinib (HER-2+) [149,150]; or ipatasertib plus paclitaxel (TNBC) [151]. Working on distinct molecular mechanisms, these chemotherapies act synergistically and eliminate most tumor cells during the initial treatment phase. However, the frequent development of drug resistance is still a major obstacle for medical personnel and BC patients. BC recurrences continued to occur steadily throughout the study period from 5 to 20 years, with risks ranging from 10% to 41% [152]. This emphasizes the urgent need for deeper insight into the molecular mechanisms leading to therapy resistance, especially chemotherapy resistance.

It is well established that EMT is not only associated with tumor metastasis and cancer cell stemness, but also with resistance to conventional therapies [6]. Chemotherapy-resistant cells treated by chemotherapeutics (e.g., oxaliplatin, 5-fluorouracil) undergo EMT, and this applies to cells treated with monoclonal antibodies (e.g., trastuzumab) as well. As outlined in previous sections, it is well established that several lncRNAs play crucial roles in the aggressiveness of BC. Next, we are going to review lncRNAs in participating signaling pathways that modulate both the aggressiveness and chemotherapy resistance of BC cells.

BC cells that undergo EMT usually show resistance to 5-fluoroutacil (5-FU) treatment [153]. Knockdown of NEAT1 sensitizes TNBC cells to chemotherapy, indicating its involvement in chemotherapy resistance [59]. Mechanistically, in-vitro and in-vivo studies have shown that NEAT1 promotes distant metastasis and 5-FU resistance through the miR-129/ZEB2 axis and miR-211/HMGA2 axis in BC [154,155].

Wnt/β-catenin signaling is one of the well-established signaling pathways that regulates EMT in various cancer types. LncRNAs regulating aggressive cytology of cancer cells via the Wnt/β-catenin signaling pathway have been reviewed before [156]. Several novel lncRNAs may regulate chemotherapy resistance through regulating Wnt/β-catenin signaling. Additionally, knockdown of lncRNA UCA1 increases the tamoxifen sensitivity of BC cells via inhibition of the Wnt/β-catenin pathway.

Differently to the lncRNAs above that were correlated with EMT, the lncRNA in the non-homologous end-joining pathway 1 (LINP1) is overexpressed in drug-resistant cells and mediates its oncogenic role in BC by decreased apoptosis-related proteins (such as caspase-8 and caspase-9), thus promoting cell growth, metastasis, and chemotherapy resistance to 5-FU and DOX in BC [157]. 5-FU and DOX are two effective and crucial chemotherapeutics against BC [158], which can interact with DNA by hindering macro-molecular biosynthesis [159].

Taken together, the studies above have revealed a new role for lncRNAs in elucidating the epigenetic mechanism of chemo-resistance. Mechanistically, most lncRNAs are recognized as involved in EMT to amplify or attenuate BC cell response to chemotherapy, and among them, Wnt/β-catenin signaling is a crucial common pathway. LncRNA-regulated apoptosis of BC cells also has an additional effect on chemotherapy resistance to 5-FU and DOX. However, more tissue samples and animal models are required to further investigate the correlation between associated lncRNA expression and patient clinicopathological features, in order to evaluate the potential of lncRNAs as independent biomarkers in BC.

## 7. LncRNAs That Function as Prognostic Biomarkers for BC Patients

### 7.1. LncRNAs as Prognostic Biomarkers for BC Patients

As we reviewed above, MALAT1 could be either a metastasis promoter or suppressor in BC patients. In terms of prognosis, current research approves of the association between over-expression of MALAT1 and poor prognosis in BC patients. Meta-analysis indicated that upregulated expression of MALAT1 in BC tissues is significantly associated with more lymph node metastasis, shorter 5-year adverse disease-free survival (DFS) and shorter overall survival (OS) [160]. Interestingly, MALAT1 was also an important pro-inflammatory factor regulating lipopolysaccharide-induced inflammatory responses in endothelial cells of BC. Li et al. found that MALAT1 was elevated in BC patients with postoperative fever, and high expression of MALAT1 predicted adverse short-term recurrence-free survival (RFS) [81]. Additionally, an upregulated MALAT1 expression level was related to positive progesterone receptor (PR) status. This might be coincident with the dual role of MALAT1 in BC [160].

Many studies have illustrated the tumor-promoting role of HOTAIR in BC progression and the association between over-expression of HOTAIR in tissues and shorter survival in BC patients [161]. Of particular note, the Kaplan-Meier survival curve showed that patients with high circulating HOTAIR expression had a worse DFS than those with low circulating HOTAIR [67]. Similarly, plasma H19 levels were significantly correlated with lymph node metastasis, and high plasma H19 levels were significantly reversed in postoperative samples [162]. In addition, analysis in the preoperative and postoperative plasma samples showed the postoperative levels of circulating GAS5 and H19 significantly decreased, and circulating GAS5 levels in the patients with a positive lymph node metastasis state decreased after surgery [163]. Therefore, circulating HOTAIR, H19 and GAS5 could be potential biomarkers for BC early screening and prognosis monitoring.

**Figure 1:**
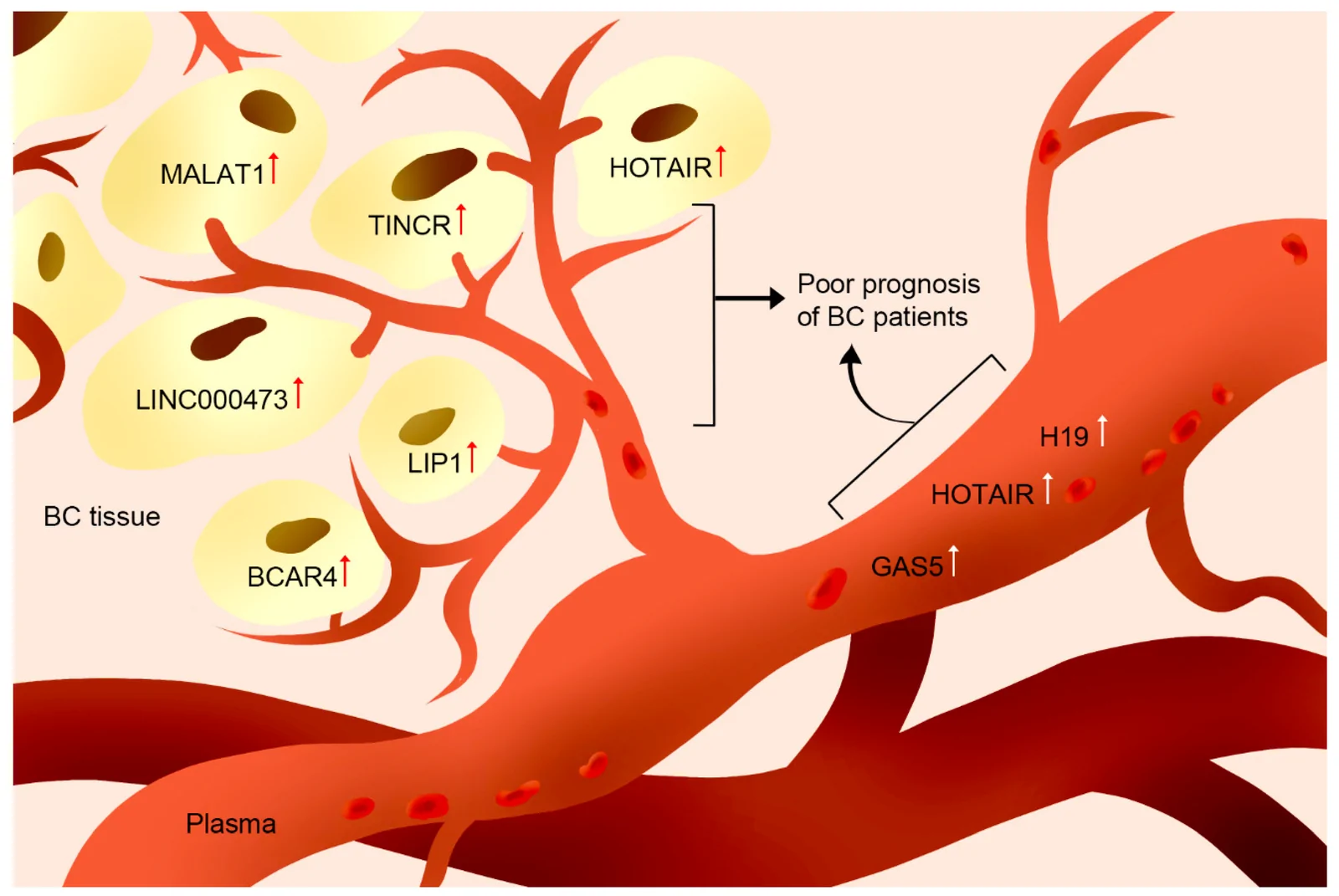
LncRNAs and prognosis of BC patients. Here both up-regulated lncRNA in BC tissue (HOTAIR, TINCR, LIP1, MALAT1, and LINC000473) and circulating lncRNA in plasma (HOTAIR, H19, and GAS5) predict poor prognosis of BC patients. The red or white up arrows respectively refer to up-regulation of the relevant lncRNA in BC patients’ tissue or plasma.

In addition, as shown in Figure 1, the expression of LINC00473 [164], TINCR, LINP1 [165] and lncRNA breast cancer anti-estrogen resistance 4 (BCAR4) [166,167] were all up-regulated in BC tissues and increased further during metastasis, and they were all associated with poor prognosis of BC patients. Of particular note, the over-expression of BCAR4 was not only negatively correlated with OS in BC patients, but was also highly correlated with the incidence of BC, so it might be applied as a major negative and unfavorable prognostic marker for BC [166,167].

### 7.2. LncRNAs as Biomarkers for Diagnosis and Prognosis of TNBC Patients

Due to the lack of effective targeted therapies and high recurrence rate in response to chemotherapy, the prognosis of TNBC is the most unfavorable among all types of BC. It is therefore urgent to identify the prognostic biomarkers for the diagnosis of TNBC, or its individualized therapy targets.

**Figure 2:**
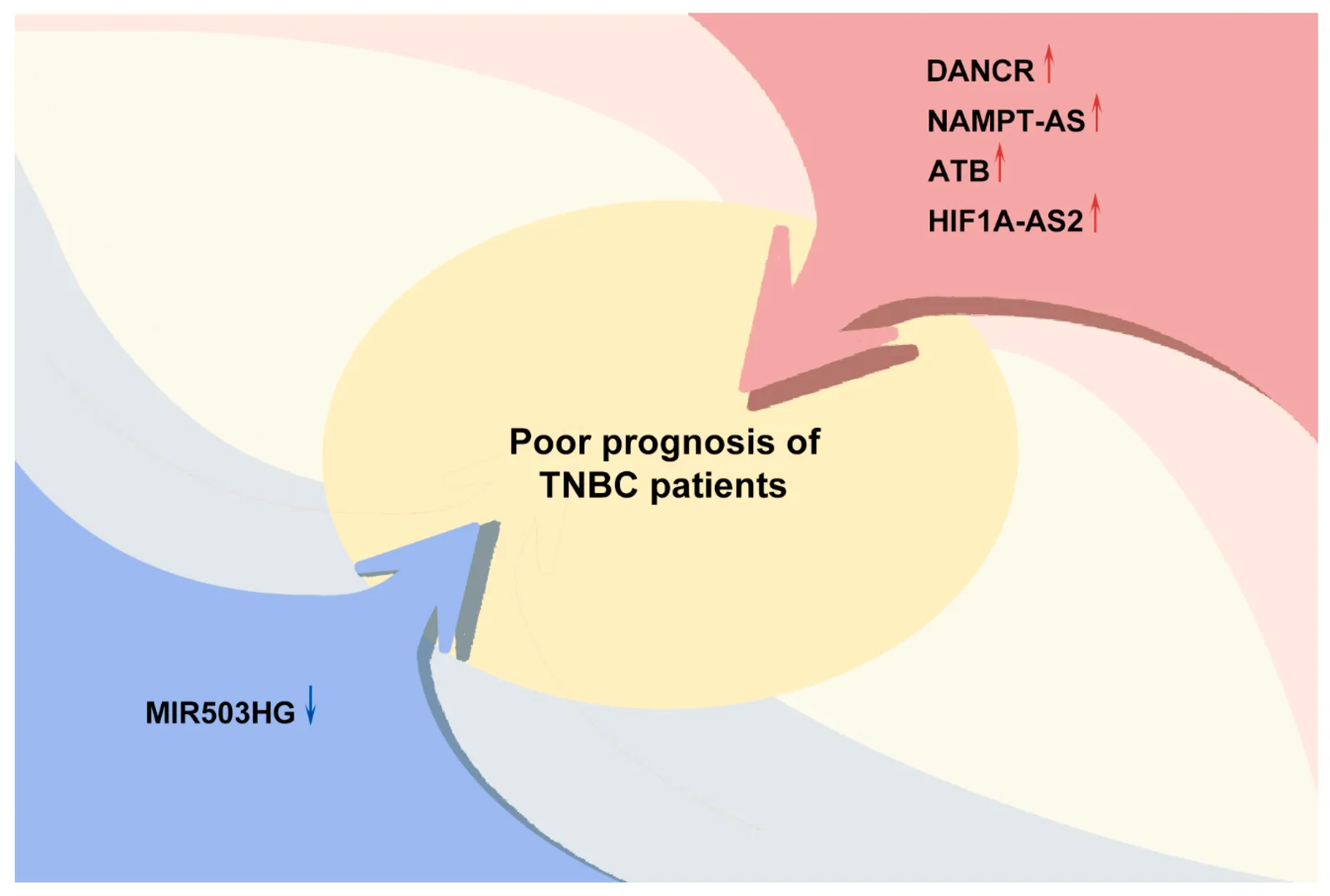
LncRNAs as biomarkers for diagnosis and prognosis of TNBC patients. Up-regulated expression of DANCR, NAMPT-AS, ATB, and HIF1A-AS2 predict poor prognosis of TNBC patients. Down-regulated expression of MIR503HG predicts poor prognosis of TNBC patients. Red up arrows refer to up-regulation of the relevant lncRNA. Blue down arrows refer to down-regulation of the relevant lncRNA.

In 2016, through detecting the expression levels of lncRNAs in TNBC and non-TNBC tissues separately, Lv et al. identified four lncRNAs (RP11-434D9.1, LINC00052, BC016831, and immunoglobulin kappa variable (IGKV)) as biomarkers to differentiate TNBC cancer from non-TNBC [168]. As shown in Figure 2, we reviewed the latest studies that revealed lncRNAs as novel prognostic biomarkers for TNBC patients in particular.

Some tumor-promoting lncRNAs in TNBC may function as biomarkers for diagnosis and prognosis of TNBC patients. Collation of research on 63 TNBC tissues showed DANCR expression was abruptly upregulated and correlated with worse OS and TNM stages [27]. By utilizing The Cancer Genome Atlas (TCGA) database and analyzing clinical TNBC samples, Zhang et al. found that the expression of nicotinamide phosphoribosyltransferase antisense RNA (NAMPT-AS) was up-regulated, which epigenetically activates NAMPT to promote tumor progression and metastasis [169]. In addition, according to Kaplan-Meier survival curve analysis, up-regulated expression levels of lncRNA-ATB, NAMPT-AS and HIF1A-AS2 are negatively correlated with OS and DFS of TNBC patients [74,169,170].

Further, some tumor-suppressive lncRNAs in TNBC may function as biomarkers for diagnosis and prognosis of TNBC patients as well. For example, the latest study observed that TNBC patients with low miR-503 host gene (MIR503HG) expression had a significantly worse prognosis compared with those with high MIR503HG expression, and low MIR503HG expression was a poor independent prognostic factor for OS in TNBC patients [171].

In summary, the clinical function of lncRNA is a topic of interest nowadays, including how they can be used for predicting prognosis or serving as therapeutic targets. However, existing research is not sufficient to support clinical applications, and the underlining clinical relevance of lncRNAs and BC still awaits for future validation. Our review aims to provide direction for further research in the future to promote better clinical practice applications.

## 8. Discussion

BC is an aggressive malignant disease in women worldwide, with a high tendency to metastasize [1]. Novel lncRNA discoveries and the increasing quality of lncRNA function studies are providing new ideas to improve understanding of the mechanism of BC pathogenesis and progression. It has been demonstrated that many lncRNAs have crucial roles in the cell proliferation of BC [172]. Despite the fact that BC is at the forefront of cancer research, very few studies had investigated the association between lncRNAs and BC distant metastasis until the last 5 years.

Here, we have reviewed the latest findings of how lncRNAs participate in BC progression, especially metastasis ability. Many researchers have tried to cast light on signaling pathways involved in the metastasis of BC. As shown in Table 1, we summarize the mechanisms of different processes that are necessary for metastasis to take place.

- LncRNAs participate in regulating the aggressiveness of BC cells (such as HOTAIR, H19, DANCR, RP1, GAS5, LNC00152, NEF, and NKILA).
- LncRNAs can regulate the stemness properties of BC cells (such as HOTAIR, H19, ES1, TUNAR, lncRNA-Hh, XIST, and FEZF1-AS1).
- LncRNAs can regulate angiogenesis of BC (such as MALAT1, MEG3, and LINC00968).

In the next section, we briefly review lncRNAs that can regulate the sensitivity to chemotherapy in vitro and in vivo, and some may function as biomarkers to diagnosis or predict prognosis for BC patients (such as HOTAIR, H19, MALAT1, TINCR, LINC00473, LINP1, BCAR4, and SNHG6).

Interestingly, we found that some particular lncRNAs have dual effects in BC, indicated by opposite previous findings and conclusions; for example, different research provided almost opposite evidence for the roles of MALAT1 in BC. Of note, there exist some differences in the materials and design of these experiments; for example, Kim et al. suggested MALAT1 was a suppressor by conducting experiments on MCF10A and MDA-MB-231 cells [104]. Arun et al. report that genetic loss or systemic knockdown of Malat1 results in slower tumor growth accompanied by significant differentiation into cystic tumors and a reduction in metastasis [83]. Additionally, Li et al. suggested MALAT1 might promote recurrence and lung metastasis of BC patients with early postoperative fever by analyzing plasma levels of MALAT1 in 258 cases of patients with primary breast carcinoma, and knock down of MALAT1 in 4T1 xenograft mice [81]. Therefore, the foremost cause of the discrepancy may be due to the tumor microenvironment (TME) and metabolism of lncRNA in vivo, and clearly further work needs to be performed to establish whether MALAT1 does have dual effects in BC.

The intimate association between EMT and CSCs has provided further insights into tumor progression and the contribution of CSCs to tumor metastasis and colonization. Interestingly, most studies on CSC and EMT center on the epigenetic differences between CSCs and non-stem cancer cells, while irreversible mutations in genes can also endow cells with metastasis ability. For example, gene-specific cell cloning in pancreatic cancer and medulloblastoma causes tumor metastasis [174,175]. This unexpected phenomenon raises a question: Is it that only CSC can lead to metastasis through genetic changes, or can non-stem cancer cells acquire tumorigenesis and metastasis at the same time via genetic changes? This question still needs in-situ cancer formation experiments to find an answer.

Autophagy is a rather conservative biological process, regulated by autophagy-related genes (ATGs) [176]. In this process, eukaryotic organelles and various substances form autophagosomes in the phospholipid bilayer; subsequently, autophagosomes go through degradation and recycling after transporting to the lysosomes. Some state that autophagy is a double-edge-sword for tumor metastasis. On the one hand, it reduces tumor necrosis induced by hypoxia, inhibits the infiltration of inflammatory cells, and thus inhibits tumor metastasis [177]. On the other hand, in the early stages of metastasis, autophagy accelerates tumor metastasis through promoting the survival of cells under metabolic stress and oxygen-deficient conditions [178]. In breast cancer pathogenesis, the correlation between lncRNAs and autophagy partly rely on complex regulation of the mTOR signaling pathway. Some lncRNA, for instance, the lncRNA NBR2 (LINC00473), functioned as a negative regulator of the mTOR pathway, and it sugnificnatly promoted autophagy. By modulaiting autopahge, NBR2 inhibits breast cancer cell tumor growth and metastasis [179].

Some consider that autophagy can promote tumor metastasis by reducing anoikis of cancer cells [180]. Anoikis refers to programmed apoptosis after separation from ECM [181]. The anoikis prevention of detached malignant cancer cells is the precondition for metastasis, and an elevated threshold for anoikis usually leads to heightened metastatic potential [182]. Currently, more and more studies are investigating the role of lncRNAs in cancer cell anoikis. For example, in thyroid carcinoma, lncRNA FOXD2 adjacent opposite strand RNA 1(FOXD2-AS1) promotes the cancer stem cell features and anoikis resistance in thyroid cancer cells via inhibiting the miR-7-5p/telomerase reverse transcriptase (TERT) axis [183]. In ovarian cancer, caspase 3/7 assay showed that MALAT1 knockdown resulted in increased anoikis [184]. Similarly, in bladder cancer, knockdown of LINC00958 attenuated resistance to anoikis [185]. However, as of the time of writing, no research has revealed how lncRNAs function in the anoikis of BC cells. We consider that this may be a promising area for future research.

In five breast cancer subtypes (luminal A, luminal B, HER2-overexpressing, basal-like, and normal-like), the pathological characteristics and prognostics exhibit significant differences [186,187]. In order to elucidate the mechanism and improve personalized clinical treatment, systematical analysis of these common and specific lncRNAs is significant.

HOTAIR acts as a vital cancer-promoting lncRNA in the BC process, and it was found to be up-regulated in the three described subtypes (ER-positive, HER2-positive and TNBC) of the disease [188,189]. After assessing the expression profile of BC subtypes, Mathias et al. found down-regulation of LINC0051 in the ER-positive subtypes and upregulation in HER2-positive and TNBC subtypes. Additionally, MEG3, LINC00152, DANCR, and MALAT1 were linked to ER-positive and TNBC subtypes. When it comes to TNBC and HER2-positive subtypes, only GAS5 was commonly expressed [188]. By conducting RNAseq of seven pairs of HER2-positive tumor vs non-tumor tissues, Yang et al. demonstrated that LOC100288637 is the highest positive correlative lncRNA with HER2, while RPL13P5 is the highest negative correlative one [190].

As is commonly known, the description of basal-like BC usually regards cell aggressiveness, stem-like phenotype, poor prognosis, and high rates of metastasis [191]. In the basal-like subtype-related network, three significantly dysregulated lncRNAs, including NEAT1, FAM83H-AS1 and XIST, were further investigated. Liu et al. demonstrated that combined use of docetaxel and carboplatin in ovarian cancer could rescue the down-regulated XIST expression [192]. Thus, we consider that XIST may be a proper example to explain drug repositioning.

One possible mechanism that links the regulation on lncRNAs to some specific subtype may be associated with specific hormone receptors. In BC cells, under hormone stimulation or deprivation, estrogen could regulate lncRNAs expression via estrogen binding or independent of estrogen binding mechanism (Apo-ER) [193]. The regulation on SNHG3, TINCR, HOTAIR, LINC00067, etc. expression is ligand dependent; the regulation on DANCR, LINC01016, DSCAM-AS1, etc. expression is ligand independent [188]. Among them, HOTAIR’s expression is induced after 17β-estradiol (E2) treatment in BC cells, indicating that HOTAIR’ expression may be associated with estrogen receptors [189]. Achievements in clinical treatment strategies have improved the survival and prognosis of BC patients. Based on the mechanisms of how lncRNAs promote tumor invasion and metastasis, researchers have begun to use drugs to regulate the expression of lncRNAs in order to impede tumor metastasis. Zhao et al. showed that a high concentration of 17b-estriol could impair the promotion of MALAT1 in the growth, invasion, and metastasis of BC cell lines by reducing lncRNA MALAT-1 expression in a dose-dependent manner [194]. In addition, the presence of acquired and de-novo resistance is still a serious concern. Many researchers have illustrated crosstalk between resistance and metastasis, which relies on EMT, plasticity acquisition, tumor heterogeneity, and so forth [195,196,197,198]. At present, research uncovering whether and how lncRNAs function during the treatment of BC is relatively sufficient, and some of them, as we discussed above, have helped to gain a better understanding of the molecular mechanisms in chemotherapy resistance. In terms of radiotherapy, the relationship between lncRNAs and radiosensitization was first demonstrated in nasopharyngeal carcinoma cells. Huang et al. showed that the lncRNA Cur enhanced radiosensitization in nasopharyngeal carcinoma cells [199]. To the best of our knowledge, in BC, HOTAIR and LINP1 were the initially investigated lncRNAs that may regulate BC radiosensitization. Knockdown of HOTAIR sensitizes BC cells to ionizing radiation through activating miR-218 [200]. Overexpression of LINP1 enhances the survival of BC cells exposed to radiation [201].

An understanding of the networks between lncRNAs, target miRNAs and genes, and BC metastasis is very useful. Besides the expression profile of lncRNAs, more functional and mechanistic investigations of lncRNA regulation of BCSC, cell autophagy and anoikis are required. Happily, lncRNAs have been in the center of public discussion since 5 years ago. This review helps to systematically understandi the molecular underpinning for finding more clinical biomarkers and treatment targets for BC in the near future.

## Figures and Tables

**Table 1:** Dual effects of lncRNAs in various stage of BC progression.

Function		LncRNA	Ensembl ID	Subcellular Localization	Signaling Axis and Pathway	References
Promote	invasion and migration of BC cells	DANCR	ENSG00000226950	Cytoplasm	DANCR/EZH2/ABCG2	[27]
					DANCR/miRNA-216a-5p	[30]
		H19	ENSG00000130600	Cytoplasm	H19/miR-152/DNMT	[36]
		HOTAIR	ENSG00000228630	Cytoplasm and nucleus	HOTAIR/P53/AKT/JNK/MMPs	[44]
					HOTAIR/AKT/mTOR	[45]
					HOTAIR/miR-20a-5p/HMGA2	[46]
		LINC00152	ENSG00000222041	Cytoplasm	LINC00152/BRCA1/PTEN	[48]
		LINC00461	ENSG00000245526	Nucleus	LINC00461/miR-30a-5p/integrin β3	[53]
		NEAT1	ENSG00000245532	Nucleus	NEAT1/miR-448/ZEB1	[58]
		LINC01857	ENSG00000224137	Nucleus	LINC01857/CREBBP/H3K27Ac and CREB1	[60]
	distant metastasis of BC cells	H19	ENSG00000130600	Cytoplasm	H19/let-7b/CYTH3/ARF	[63]
		HOTAIR	ENSG00000228630	Cytoplasm and nucleus	TGF-β1/CDK5/HOTAIR/H3K27	[69]
		HIF1A-AS2	ENSG00000258667	Cytoplasm	unclear	[173]
		MALAT1	ENSG00000251562	Nucleus	MALAT1/miR-1/slug	[79]
					MALAT1/miR-129-5p	[84]
	stemness of BC cells	TUNAR	ENSG00000250366	Cytoplasm	TUNAR/SOX2	[131]
		LINC00511	ENSG00000227036	Cytoplasm	LINC00511/miR-185-3p/E2F1/Nanog	[135]
		lncRNA-Hh			lncRNA-Hh/GAS1/SOX2/OCT4	[133]
		FEZF1-AS1	ENSG00000230316	Nucleus	FEZF1-AS1/miR-30a/Nanog	[134]
		FGF13-AS1	ENSG00000226031	Nucleus	FGF13-AS1/IGF2BPs/Myc	[136]
		ES1	ENSG00000226673	Nucleus	ES1/Oct4/Sox2/miR-302	[132]
		H19	ENSG00000130600	Cytoplasm	H19/let-7/HIF-1/PDK1	[139]
	BC angiogenesis	MALAT1	ENSG00000251562	Nucleus	MALAT1/miR-145/VEGF	[144]
Inhibit	invasion and migration of BC cells	GAS5	ENSG00000234741	Cytoplasm and nucleus	GAS5/miR-21/RISC	[87]
					GAS5/miR-196a-5p/FOXO1/PI3K/AKT	[88]
		MT1JP	ENSG00000255986	Cytoplasm	MT1JP/miR-24-3p/Wnt/β-catenin	[156]
		NKILA	ENSG00000278709	Cytoplasm	NKILA/NF-ĸB/MMPs	[97]
		NEF			NEF/miRNA-155	[94]
		LET			unclear	[98]
		TFAP2A-AS1			TFAP2A-AS1/miR-933/SMAD2	[99]
		LncKLHDC7B			unclear	[100]
	distant metastasis of BC cells	MALAT1	ENSG00000251562	Nucleus	PTEN/microRNA/MALAT1	[106]
					MALAT1/YAP-TEAD	[104]
		MEG3	ENSG00000214548	Nucleus	miR-506/SP3/SP1/DNMT1/MEG3	[108]
		NLIPMT		Cytoplasm	NLIPMT/GSK3β and EMT proteins	[112]
		XIST	ENSG00000229807	Nucleus	XIST/MSN/c-Met	[122]
					XIST/miR-155/CDX1	[120]
	stemness of BC cells	XIST	ENSG00000229807	Nucleus	XIST/MSN/EMT proteins	[122]
	BC angiogenesis	MEG3	ENSG00000214548	Nucleus	MEG3/AKT signaling	[146]
