# Correction: Huang et al. Non-Coding RNA: Dual Effects on Breast Cancer Metastasis and Clinical Applications. *Cancers* 2019, *11*, 1802

**DOI:** 10.3390/cancers18152388

**Published:** 2026-07-24

**Authors:** Qi-Yuan Huang, Guo-Feng Liu, Xian-Ling Qian, Li-Bo Tang, Qing-Yun Huang, Li-Xia Xiong

**Affiliations:** 1 Department of Pathophysiology, Basic Medical College, Nanchang University, Nanchang 330006, China; 6301616056@email.ncu.edu.cn (Q.-Y.H.); 6302615051@email.ncu.edu.cn (X.-L.Q.); 401442718029@email.ncu.edu.cn (Q.-Y.H.); 2 Second Clinical Medical College, Nanchang University, Nanchang 330006, China; 3 First Clinical Medical College, Nanchang University, Nanchang 330006, China; 6302615053@email.ncu.edu.cn; 4 Jiangxi Province Key Laboratory of Tumor Pathogenesis and Molecular Pathology, Nanchang 330006, China

In the original publication [1], there were several references cited that have been retracted or identified as problematic. Corrections have been made as follows:

**1. Deleted References**

The authors have deleted the following retracted or problematic references: Refs. [76,91,92,137,157,162,163]. Along with these removals, the corresponding descriptions, paragraphs, or entire subsections regarding *lncRNA RP1*, gastric cancer relevance in *MT1JP*, certain stemness-related mechanisms, *LINC00968*-mediated drug resistance, and *TINCR* in trastuzumab resistance have been completely removed from the main text and Table 1.

**2. Replaced References**

The authors have replaced the following problematic references with more appropriate or recent articles to maintain scientific accuracy:

Ref. [46] has been replaced with a more appropriate article [2], and the text was revised to focus on the *HOTAIR/miR-204/FAK* axis in TNBC.

Ref. [113] has been replaced with a more recent review article [3].

Ref. [186] has been replaced with a relevant review [4], with the text adjusted accordingly.

With this correction, the order of some references and corresponding rows in Table 1 have been adjusted accordingly to ensure consistency.

The correction does not include referenced data, and the scientific conclusions have not been changed. This correction was approved by the Academic Editor. The original publication has also been updated.
